# A Pharmacokinetic Evaluation of a Pectin-Based Oral Multiparticulate Matrix Carrier of Carbamazepine

**DOI:** 10.1155/2021/5527452

**Published:** 2021-07-03

**Authors:** Seth Kwabena Amponsah, Simon Yeboah, Kennedy Kwami Edem Kukuia, Benoit Banga N'guessan, Ofosua Adi-Dako

**Affiliations:** ^1^Department of Medical Pharmacology, University of Ghana Medical School, Accra, Ghana; ^2^Department of Pharmacology and Toxicology, School of Pharmacy, University of Ghana, Accra, Ghana; ^3^Department of Pharmaceutics and Microbiology, School of Pharmacy, University of Ghana, Accra, Ghana

## Abstract

**Background:**

Carbamazepine is a drug used in the treatment of neurological disorders such as epilepsy. However, due to its erratic absorption, oral bioavailability is often poor. There is, therefore, the need to develop alternative formulations for carbamazepine with better pharmacokinetic characteristics.

**Aim:**

The aim of this study was to formulate an oral modified-release multiparticulate matrix of carbamazepine from cocoa pod husk (CPH) pectin and evaluate the pharmacokinetic profile of this formulation using *in vitro* and *in vivo* models.

**Methods:**

CPH pectin was extracted from cocoa pod husks with hot aqueous and citric acid solutions. Oral multiparticulate carbamazepine matrices were formulated from CPH pectin cross-linked with calcium. The formulation was evaluated for carbamazepine content and release profile *in vitro*. For *in vivo* pharmacokinetic profile estimation, rats were put into 4 groups of 5 animals each to receive carbamazepine multiparticulate matrix formulations A and B, carbamazepine powder, and Tegretol CR^®^. Animals in each group received 200 mg/kg of each drug via the oral route. Maximum plasma concentration (*C*_max_), area under the concentration-time curve (AUC), elimination rate constant (*K*_*e*_), and terminal half-life (*t*_1/2_) of the formulations were estimated by noncompartmental analysis.

**Results:**

The pectin extraction from fresh cocoa pod husks using hot aqueous and citric acid solutions gave pectin yields of 9.63% and 11.54%, respectively. The drug content of carbamazepine in CPH pectin formulations A and B was 95% and 96%, respectively. There was controlled and sustained release of carbamazepine for both formulations A and B *in vitro*. AUC_0⟶36_ (176.20 ± 7.97 *µ*g.h/mL), *C*_max_ (8.45 ± 0.71 *μ*g/mL), *T*_max_ (12 ± 1.28 h), and *t*_1/2_ (13.75 ± 3.28 h) of formulation A showed a moderately enhanced and comparable pharmacokinetic profile to Tegretol CR^®^ (AUC_0⟶36_: 155 ± 7.15 *µ*g.h/mL, *C*_max_: 8.24 ± 0.45 *μ*g/mL, *T*_max_: 8.0 ± 2.23 h, and *t*_1/2_: 13.51 ± 2.87 h).

**Conclusion:**

Findings from the study suggest that formulations of CPH pectin had the potential to control and maintain therapeutic concentrations of carbamazepine in circulation over a period of time in the rat model.

## 1. Introduction

Epilepsy is a common neurological disorder characterized by unprovoked seizures. In 2015, among other selected neurological diseases, epilepsy was found to be the cause of more than 10 million “disability-adjusted life years” [1]. The goal in drug management of epilepsy is to ensure lasting control of seizures and reduced drug-related side effects. Often, patient noncompliance presents a challenge in attaining this goal. Moreover, immediate release formulations of antiepileptic drugs have shown wide variations in drug plasma concentrations that may result in untoward clinical effects [2–5]. Therefore, reduction in drug dosing frequency via the use of controlled release formulations could be one of the effective means in improving patient compliance and therapeutic effect [2, 5].

Carbamazepine, an antiepileptic drug, is a BCS Class II drug with poor aqueous solubility, erratic absorption, and variable bioavailability [6, 7]. The poor dissolution rate and absorption of carbamazepine could lead to low plasma concentrations which could result in therapeutic failure [2, 5]. Several approaches have been proposed to improve the bioavailability of carbamazepine. Some of the methods include novel drug delivery strategies with the use of extended-release formulations [8]. Extended-release formulations improve patient compliance, reduce potential adverse effects, and enhance therapeutic effect of drugs [2, 8].

Previous reports suggest that cocoa pod husk (CPH) pectin-based matrices are useful in enhancing bioavailability and release profiles of drugs [9]. Pectin is safe, biocompatible, biodegradable, and economical [10, 11]. Carbamazepine is relatively inexpensive compared to newer antiepileptic drugs such as gabapentin and topiramate [5]; hence, it is the drug of choice for many patients with epilepsy in resource-poor settings. There is, however, paucity of data on the role of CPH pectin-based drug delivery systems in the release and absorption of carbamazepine. The aim of the current study was to investigate the pharmacokinetic profile of formulated CPH pectin-based oral multiparticulate matrix of carbamazepine.

## 2. Methods

### 2.1. Materials

Carbamazepine powder (100.1%) was purchased from Kinapharma Limited, Ghana. Cocoa pods were gifts from Cocoa Research Institute of Ghana (CRIG), Tafo, Ghana. Tegretol 200 CR® tablets (Novartis Pharmaceuticals UK Limited, London) were purchased from Kinapharma Limited, Ghana. Hydroxypropyl methylcellulose (HPMC) was a gift from Ernest Chemists Limited (Ghana). Ethanol, citric acid, acetone, and other reagents used were purchased from Merck (Darmstadt, Germany).

### 2.2. Collection and Extraction of CPH Pectin

Ripe cocoa pods were obtained from the Cocoa Research Institute of Ghana (CRIG), Tafo, Ghana. To avoid pigmentation, fresh whole pod husks were peeled. The pulp and seeds were then removed from pods. With a mechanical blender, the peeled husks were minced. Hot aqueous citric acid (4% w/v) and hot aqueous extractions of the minced husks were done as previously described [12]. The extracts were referred to as citric acid-soluble pectin (CSP) and hot water-soluble pectin (HSP).

### 2.3. Preparation of CPH Pectin-Based Oral Multiparticulate Matrix of Carbamazepine

The HSP-based matrix of carbamazepine granules of a total weight of 12 g was prepared, each 400 mg containing 100 mg carbamazepine. The stipulated amounts of excipients were weighed and with the exception of HSP were mixed by geometric dilution. In order to form a viscous dispersion, the weighed HSP was dispersed in adequate amount of hot water. The mixed powders were added gradually to the dispersion by geometric dilution to form a wet mass. The wet powdered mass was taken through a #20 sieve, and the granules were made dry in an oven (at 40°C) for 1.5 h. The same procedure was repeated for CSP.

The various formulations of CPH pectin-based oral multiparticulate matrices of carbamazepine are shown in Table 1.

### 2.4. Moisture Content of CPH Pectin-Based Carbamazepine Multiparticulate Matrices

The moisture content of the formulations (A, B, and C) was estimated by weighing 1 g of prepared matrices into Petri dishes and drying in hot-air oven at 105°C. The moisture content was computed as a ratio of the weight of moisture lost to the initial weight of sample and expressed as a percentage [12–14].

### 2.5. Determination of Swelling Indices of Pectins

The swelling indices of the pectins (HSP-based matrix and CSP matrix) were done as described elsewhere [12]. One gram of the sample was weighed into a measuring cylinder and, the initial volume occupied in 0.1 N HCL and then phosphate buffer (pH 6.8); both volumes were recorded (*V*_1_). The final volume for each upon standing was recorded (*V*_2_). The swelling capacity was calculated as follows: (*V*_2_/*V*_1_) × 100.

### 2.6. Carbamazepine Content in Formulations

The content of carbamazepine in the formulation corresponding to 400 mg (and which is equivalent to ∼100 mg carbamazepine) of the granular matrix was determined using reverse-phase high-performance liquid chromatography (RP-HPLC), as previously described [15].

### 2.7. *In Vitro* Release of Formulated Carbamazepine Multiparticulate Matrices in Simulated Intestinal Fluid

The release of carbamazepine from the CPH pectin-based multiparticulate matrices in simulated intestinal fluid was conducted using a USP Dissolution Tester Apparatus I (Lid-8 Dissolution Tester, Vanguard Pharmaceuticals Machinery Inc., USA). Multiparticulate matrices with weight 400 mg were placed in a basket and further immersed in a vessel containing 900 ml of phosphate buffer (pH 6.8). The buffer served as dissolution media and was maintained at 37 ± 0.5°C and 50 rpm. Afterwards, 5 ml aliquots was drawn and replaced with the same volume of fresh dissolution media at specific time intervals. The absorbance of the samples after dilution was at 284 nm using a UV Spectrophotometer (Merck, Darmstadt, Germany). Triplicate determinations were done [16].

### 2.8. *In Vivo* Pharmacokinetic Evaluation of Carbamazepine Multiparticulate Matrices

Male Sprague Dawley (SD) rats that weighed between 150 g and 200 g and aged between 6 weeks and 8 weeks were obtained from the Animal Experimentation Unit, Department of Medical Microbiology, University of Ghana Medical School. Rats were housed in stainless steel cages with a minimum space of 2 cubic feet (61 cm × 31 cm) with soft wood shavings as bedding. Rat chow was normal pellet diet (AGRIMAT, Kumasi, Ghana). The SD rats were given water *ad libitum* and maintained under optimal laboratory conditions (relative humidity 60–70%, temperature 25 ± 1°C, and 12-hour light-dark cycle). To prevent contamination, feeding and water troughs were regularly cleaned. The SD rats were made to acclimatize to this environment for 14 days before start of experiment.

Randomly, the SD rats were put into 4 groups consisting of 5 rats each. Prior to administration of treatments, the SD rats were fasted overnight. Group 1 received formulation A (100 mg carbamazepine, 200 mg HSP, 50 mg calcium chloride, and 50 mg HPMC), Group 2 received formulation B (100 mg carbamazepine, 200 mg CSP pectin, 50 mg calcium chloride, and 50 mg HPMC), animals in Group 3 were given pure carbamazepine powder, and Group 4 were given Tegretol CR^®^ tablets. A dose of 200 mg/kg of carbamazepine of the respective formulations was given via the oral route to rats in each group. Treatments were administered every 12 h. After administration of the second dose of each treatment on the second day, the tails of rats were snipped and samples of blood collected into Ethylenediaminetetraacetic acid (EDTA) tubes at times 0.5, 1, 2, 8, 12, 24, and 36 h [17]. To obtain plasma, blood samples were centrifuged at 2500 rpm for 10 min. Carbamazepine in plasma was analysed using HPLC as described by Mowafy et al. [18].

### 2.9. Ethical Issues

The Ethics and Protocol Review Committee of the College of Health Sciences, University of Ghana, approved this research. The protocol identification number is CHS-Et/M.8-5.14/2019-2020.

### 2.10. Data Analysis

For comparison of more than two independent sample means, one-way analysis of variance (ANOVA) was used. Noncompartmental analysis was used to estimate pharmacokinetic parameters of carbamazepine in the 4 groups. Peak plasma drug concentration (*C*_max_) and the time to achieve this peak (*T*_max_) of carbamazepine were obtained from concentration-time curves. Linear regression analysis of the terminal-linear part of the log plasma concentration-time curves was used to estimate elimination rate constant (*K*_*e*_). The formula *t*_1/2_ = 0.693 Ke^−1^ was used to compute the elimination half-life (*t*_1/2_). Linear trapezoidal rule was used to estimate the area under the concentration-time curve (AUC).

## 3. Results

### 3.1. Moisture Content and Percentage Yield of Extracted Cocoa Pod Husk Pectin

The hot water-soluble pectin (HSP) had a moisture content of 0.06% and a yield of 9.63%, while the citric acid-soluble pectin (CSP) had a moisture content of 0.04% and a yield of 11.54%. The swelling indexes of HSP- and CSP-based pectins in 0.1 N HCL were 150 and 120, respectively. In phosphate buffer (pH = 6.8), the swelling indexes for HSP- and CSP-based pectins were 170 and 130, respectively.

### 3.2. Content Analysis of Formulated Carbamazepine Multiparticulate Matrices

Analyses of the 3 new formulations of cocoa pod husk pectin-based modified-release formulations of carbamazepine are shown in Table 2.

### 3.3. *In Vitro* Release of Formulated Carbamazepine Multiparticulate Matrices from Simulated Intestinal Fluid

**Figure 1:**
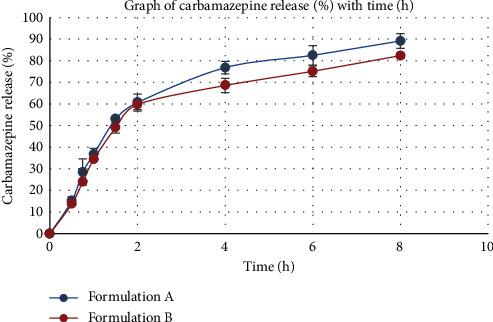
*In vitro* release of the two formulated carbamazepine multiparticulate matrices from simulated intestinal fluid. Formulation A = 100 mg carbamazepine, 200 mg HSP pectin, 50 mg calcium chloride, and 50 mg HPMC. Formulation B = 100 mg carbamazepine, 200 mg CSP pectin, 50 mg calcium chloride, and 50 mg HPMC.

Figure 1 shows an initial burst or accelerated release after which there is a slow release of drug indicative of a biphasic release pattern useful for the maintenance of sustained drug concentration. There was ∼80% of carbamazepine released within 8 hours for both formulations A and B. The *in vitro* release of the multiparticulate matrices revealed that formulations A and B had similar release profiles and exhibited sustained release characteristics, and as such, formulations A and B were further investigated in the rat model.

### 3.4. Concentration-Time Curves and Pharmacokinetic Parameters of Carbamazepine among Rats in Treatment Groups

**Figure 2:**
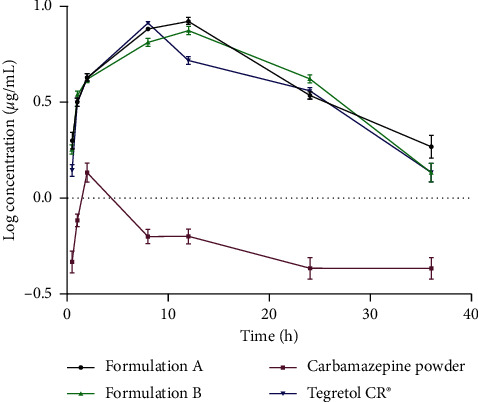
Semilogarithm concentration-time curves of carbamazepine for the 4 treatment groups (5 rats per group).

The concentration-time curves for SD rats in the 4 groups, Group 1 (received formulation A), Group 2 (received formulation B), Group 3 (given pure carbamazepine powder), and Group 4 (given Tegretol CR^®^), are shown in Figure 2. By visual inspection, the curve of rats administered with formulation A had the highest peak, followed by Tegretol CR^®^ and formulation B. Rats administered with carbamazepine powder had the lowest peak.

Pharmacokinetic parameters of carbamazepine in the 4 treatment groups are summarized in Table 3.

From Table 3, the relative bioavailabilities (Rel F) for formulations A and B were ∼114% and 104%, respectively.

## 4. Discussion

Carbamazepine remains one of the most widely used drugs in the management epilepsy and other neurological disorders. However, conventional dosage forms (immediate release) of carbamazepine are plagued with erratic absorption and variable bioavailability. In the current study, CPH pectin-based multiparticulate matrices of carbamazepine were evaluated *in vitro* and in Sprague Dawley rats.

Three CPH pectin-based oral multiparticulate matrices of carbamazepine were formulated to achieve controlled release. Several techniques and solvents such as hydrochloric acid, nitric acid, citric acid, and water have been used successfully in extraction of CPH pectins and have produced varying degrees of yield and pectin qualities [19–21]. Hot citric acid solution and hot aqueous extraction methods were employed in this study because of known safety and environmental friendliness. Citric is an organic acid which is a safe food additive and its use in extraction of CPH pectins often curtails corrosive effects that conventional acids (such as hydrochloric acid and nitric acid) may have. The CPH pectin yield obtained from extractions were 9.63% and 11.54% for hot water-soluble and citric acid-soluble pectins, respectively. Previous studies conducted by Priyangini et al. [22] reported a 4.2% and Vriesmann et al. [23] reported a 10.1% yield of the citric acid solution extract, respectively. It is noteworthy that the extraction methods employed could influence the yield and formulation properties of pectins and thereby affect drug release [24, 25]. Future studies could optimize extraction yields via increasing temperature, extraction time, and repeated extraction to exhaustion [23].

Moisture content of pharmaceutical powders are usually monitored and controlled. Usually microbes could multiply in aqueous media resulting in degradation of drug. The moisture content of both hot water-soluble and citric acid-soluble pectins were low (0.06% and 0.04%, respectively). The formulated CPH pectin-based multiparticulate matrices may have the added advantage of enhanced stability due to low moisture content [26]. Low moisture content of pectins does not only protect drug formulations from microbial degradation, but this property also improves mechanical properties of drugs [27].

The swelling properties of HSP- and CSP-based matrices were investigated in acidic and basic media. The swelling indices of the HSP was 150 in 0.1 N HCl and 170 in phosphate buffer (pH of 6.8). CSP had an index of 120 in 0.1 N HCL and 130 in phosphate buffer (pH of 6.8). Previous reports indicate that CPH pectin can swell to varying extents in various media [12]. The swelling characteristics exhibited indicate that CPH pectin could function as a matrix or release modifier in controlled-release formulations, as swelling is an important mechanism in diffusion controlled release in drug delivery [28].

The carbamazepine content of formulations A and B was 95% and 96%, respectively. These values fell within the stipulated range for drug formulation [29]. However, formulation C had carbamazepine content of 89.5% and did not merit any further investigation.

The *in vitro* study of the two multiparticulate matrices (formulations A and B) was performed in a simulated gastrointestinal fluid to mimic and make a prediction of the release of carbamazepine *in vivo* [30, 31]. Formulations A and B exhibited sustained *in vitro* release profiles.

The CPH pectin-based modified-release formulations of carbamazepine were prepared as oral multiparticulate matrices because of potential benefits in drug delivery. Oral multiparticulate matrix delivery system is known to possess a higher surface area, increased bioavailability, and a possibly longer residence time in the gastrointestinal tract [32]. Such granular matrix systems have a low risk of dose dumping, have a predictable distribution and gastrointestinal transport, and are versatile enough to be tailored to suit various drug release patterns [33, 34].

The pectin-based multiparticulate matrix swells up when in contact with aqueous fluids initiating a rapid release; subsequently, there is diffusion of the drug accompanied with a slow and prolonged release [35]. A biphasic drug release pattern is useful for providing an initial rapid release, and then maintaining a slow release of drug for prolonged periods [36] was observed with both formulations A and B. Investigations into the use of multilayer dosage forms, compressed core tablets, and versatile minitablets in achieving a biphasic drug release have been documented. The strategy employed in this regard could include both immediate and sustained release component. The immediate release component provides the loading dose and the sustained release portion maintains the effective plasma concentration over a prolonged duration of time [37, 38]. In the current study, the adoption of a simple formulation design and strategy with the use of a pectin-based multiparticulate matrix system exhibited a biphasic release pattern which elicits controlled and sustained therapeutic drug concentrations. [39]. This formulation design presents as a suitable and cost-effective alternative.

Modification of pectin matrices in the formulation of composites with other polymers or in combination with other ions allows for tailor-made materials to suit drug delivery needs. In essence, the composite has the benefits of the parent polymers and is able to overcome the limitations of the parent polymers with the creation of beneficial novel drug delivery systems.

The CPH pectin matrices of carbamazepine were formulated to provide controlled release in the gastrointestinal tract. However, this sometimes presents a challenge since CPH pectin is a highly hydrophilic polymer with the likelihood of untimely drug release due to its high gastrointestinal tract solubility. The aqueous solubility of CPH pectin was reduced by crosslinking with calcium. Calcium ions in the pectin matrix reduce drug release from the hydrophilic pectin [40–42]. Crosslinking with calcium ions inhibits drug release by reducing the dissolution and swelling of the pectin matrix. In effect, there is inhibition in dissolution and swelling of CPH pectin with incorporation of calcium. Furthermore, HPMC had the ability to strengthen the polymer composite matrix and ensured an enhanced gel strength of the carbamazepine-calcium matrix [43, 44].

The CPH pectin composites (calcium and HPMC) were effective in controlling the release of carbamazepine in the gastrointestinal tract. The newly formulated CPH pectin-based multiparticulate matrices of carbamazepine (formulations A and B) showed significant improvement in pharmacokinetic profiles (*T*_max_, *C*_max_, AUC_0⟶36_, *K*_*e*_,  and *t*_1/2_) compared with the carbamazepine powder. Although both formulations A and B exhibited a modestly enhanced pharmacokinetic profile over Tegretol CR^®^, the differences in pharmacokinetic data did vary significantly (*p* < 0.05). It was evident in the current study that the modified-release formulation (multiparticulate matrices) played a role in enhancing the pharmacokinetic parameters of the poorly soluble drug, carbamazepine.

Data from current study showed that peak carbamazepine concentrations (*C*_max_) did not differ significantly between formulation A vs. formulation B (*p*=0.3928), formulation A vs. Tegretol CR^®^(*p*=0.7895), and formulation B vs. Tegretol CR^®^(*p*=0.4618). The higher serum concentration of formulation A (8.45 ± 0.71 *µ*g/mL) than formulation B (7.74 ± 0.70 *µ*g/mL) may suggest a more controlled absorption rate exhibited by the citric acid solution extract- (CSP-) based matrix of formulation B than the hot water-soluble extract- (HSP-) based matrix of A [45]. Additionally, the maximum serum carbamazepine concentrations (*C*_max_) obtained for formulations A and B were comparable to that of the standard drug Tegretol CR^®^ [2, 46].

A comparison of time to achieve peak carbamazepine concentration (*T*_max_) between formulation A and formulation B (*p*=0.1502), formulation A and Tegretol CR^®^(*p*=>0.9999), and formulation B and Tegretol CR^®^(*p*=>0.9999) did not differ significantly. The time for carbamazepine to achieve peak concentration was longer for the CPH pectin matrix modified-release formulations (formulation A: 12 ± 1.28 *µ*g/mL and formulation B: 12 ± 1.43 *µ*g/mL) as compared to Tegretol CR^®^ (8 ± 2.23 *µ*g/mL). The matrix formulations demonstrated a prolonged release of carbamazepine, which is known to enhance patient compliance and reduce frequency of dosing [47, 48]. Even though the difference in time to achieve peak concentration between formulation A, formulation B, and Tegretol CR^®^ (the standard drug) was not significant (*p* < 0.05), the findings suggest a modest improvement in the release profiles of the CPH pectin-based carbamazepine matrix formulations.

The total drug exposure (AUC) did not differ significantly between formulation A vs. formulation B (*p*=0.9600), formulation A vs. Tegretol CR^®^(*p*=0.1090), and formulation B vs. Tegretol CR^®^(*p*=0.1589). From Table 3, AUC_0⟶36_ indicated a higher total drug exposure for formulation A (176.20 ± 7.97 *μ*g^*∗*^h/mL) and formulation B (161 ± 4.284 *μ*g^*∗*^h/mL) than Tegretol CR^®^ (155 ± 7.15 *μ*g^*∗*^h/mL). Additionally, the relative bioavailabilities of the CPH pectin-based matrices A and B was ∼114% and ∼104%, respectively. This is indicative of the ability of the modified-release matrix formulations to slightly extend drug exposure in the current animal model study and corroborates other studies that report the same [3, 33, 49–52].

Data from current study showed that the elimination half-lives (*t*_1/2_) of formulations A and B were 13.75 ± 3.28 h and 13.23 ± 1.90 h, respectively. The prolonged effect of both CPH pectin-based modified-release matrices of carbamazepine compared with carbamazepine powder may aid in reduction of dosing frequency [8] and further improve patient compliance.

## 5. Conclusion

*In vivo* kinetic assessment of formulated CPH pectin-based multiparticulate matrix of carbamazepine showed that this formulation had relatively high peak plasma concentration, prolonged total drug exposure, and long half-life.

## Figures and Tables

**Table 1:** Composition of CPH pectin-based carbamazepine multiparticulate matrices.

Formulation	Carbamazepine (mg)	HSP (mg)	CSP (mg)	Calcium chloride (mg)	HPMC (mg)
A	100	200	—	50	50
B	100	—	200	50	50
C	100	200	—	—	100

CPH = cocoa pod husk; HPMC = hydroxypropyl methylcellulose.

**Table 2:** Content analysis of CPH pectin-based modified-release formulation of carbamazepine.

Sample	Carbamazepine content (%)
Formulation A	95.0
Formulation B	96.0
Formulation C	89.5

Formulation A = 100 mg carbamazepine, 200 mg hot water-soluble pectin, 50 mg calcium chloride, and 50 mg HPMC; formulation B = 100 mg carbamazepine, 200 mg citric acid-soluble pectin, 50 mg calcium chloride, and 50 mg HPMC; and formulation C = 100 mg carbamazepine, 200 mg CPH pectin, and 100 mg HPMC.

**Table 3:** Pharmacokinetic parameters of carbamazepine in the 4 treatment groups.

Parameter	Treatment group (mean (standard deviation))				*p* value
	Formulation A	Formulation B	Carbamazepine powder	Tegretol CR^®^	
*T*_max_ (h)	12 (1.28)	12 (1.43)	2 (0.56)	8 (2.23)	0.007
*C*_max_ (*µ*g/mL)	8.45 (0.71)	7.74 (0.70)	1.45 (0.45)	8.24 (0.45)	0.001
AUC_0⟶36_ (*μ*g^*∗*^h/mL)	176.20 (7.97)	161 (4.24)	22.05 (1.64)	155 (7.15)	<0.0001
*K*_*e*_ (h^−1^)	0.05 (0.01)	0.04 (0.01)	0.08 (0.03)	0.05 (0.02)	0.242
*t*_1/2_ (h)	13.75 (3.28)	13.23 (1.90)	8.44 (6.97)	13.51 (2.87)	0.691

Formulation A = 100 mg carbamazepine, 200 mg CPH pectin, 50 mg calcium chloride, and 50 mg HPMC; formulation B = 100 mg carbamazepine, 200 mg CPH pectin, 50 mg calcium chloride, and 50 mg HPMC.

## Data Availability

Data used to support the findings of this study are available from the corresponding author upon request.

## References

[1] WHO (2019). *Epilepsy: a Public Health Imperative*.

[2] Koester L. S., Dalla Costa T., Bassani V. L. (2004). Pharmacokinetics of carbamazepine from extended release dosage forms: bioavailability/bioequivalence and in vitro-in vivo correlation studies. *Journal of Drug Delivery Science and Technology*.

[3] Barakat N. S., Elbagory I. M., Almurshedi A. S. (2008). Formulation, release characteristics and bioavailability study of oral monolithic matrix tablets containing carbamazepine. *AAPS PharmSciTech*.

[4] Verrotti A., Iapadre G., Di Donato G. (2019). Pharmacokinetic considerations for anti-epileptic drugs in children. *Expert Opinion on Drug Metabolism & Toxicology*.

[5] Li H., Zhang M., Xiong L., Feng W., Williams R. O. (2020). Bioavailability improvement of carbamazepine via oral administration of modified-release amorphous solid dispersions in rats. *Pharmaceutics*.

[6] Chonkar A., Udupa N., Venkata Rao J., Hardikar S. (2017). Development and evaluation of orodispersible tablet of microwave generated Co-processed material of carbamazepine. *Advanced Science Letters*.

[7] Cvetkovski A. (2018). Increasing water solubility, the prerequsite for improvement of bioavailability. *Acta Medica Balkanica, International Journal of Medical Sciences*.

[8] Leppik I. E., Hovinga C. A. (2013). Extended‐release antiepileptic drugs: a comparison of pharmacokinetic parameters relative to original immediate‐release formulations. *Epilepsia*.

[9] Adi-Dako O., Ofori-Kwakye K., Amponsah S. K. (2018). Potential of cocoa pod husk pectin-based modified release capsules as a carrier for chronodelivery of hydrocortisone in sprague-dawley rats. *Journal of Drug Delivery*.

[10] Espitia P. J. P., Du W. X., Avena-Bustillos R. D. J., Ferreira Soares N. D. F., McHugh T. H. (2014). Edible films from pectin: physical-mechanical and antimicrobial properties-a review. *Food Hydrocolloids*.

[11] Martău G. A., Mihaela M., Vodnar D. C. (2019). The use of chitosan, alginate, and pectin in the biomedical and food sector—biocompatibility, bioadhesiveness, and biodegradability. *Polymers*.

[12] Adi-Dako O., Ofori-Kwakye K., Frimpong Manso S. (2016). Physicochemical and antimicrobial properties of cocoa pod husk pectin intended as a versatile pharmaceutical excipient and nutraceutical. *Journal of Pharmaceutics*.

[13] British Pharmacopoeia (2007). *The British Pharmacopoeia. 2007 (Electronic Version)*.

[14] British Pharmacopoeia (2016). *British Pharmacopoeia*.

[15] Adi-Dako O., Ofori-Kwakye K., Oppong Bekoe S., Appiah E., Peprah P. (2017). Novel HPLC analysis of hydrocortisone in conventional and controlled-release pharmaceutical preparations. *Journal of Pharmaceutics*.

[16] Adi-Dako O., Ofori-Kwakye K., Oppong Bekoe S., Okyem S., El Boakye-Gyasi M. (2017). In vitro evaluation of cocoa pod husk pectin as a carrier for chronodelivery of hydrocortisone intended for adrenal insufficiency. *Journal of Drug Delivery*.

[17] Dankyi B. O., Kwabena Amponsah S., Lovia Allotey-Babington G. (2020). Chitosan coated hydroxypropylmethyl cellulose microparticles of levodopa (and carbidopa): in vitro and rat model kinetic characteristics. *Current Therapeutic Research*.

[18] Mowafy H. A., Alanazi F. K., El Maghraby G. M. (2012). Development and validation of an HPLC–UV method for the quantification of carbamazepine in rabbit plasma. *Saudi Pharmaceutical Journal*.

[19] Fishman M. L., Chau H. K., Kolpak F., Brady J. (2001). Solvent effects on the molecular properties of pectins. *Journal of Agricultural and Food Chemistry*.

[20] Shafie M. H., Yusof R., Gan C. Y. (2019). Deep eutectic solvents (DES) mediated extraction of pectin from Averrhoa bilimbi: optimization and characterization studies. *Carbohydrate Polymers*.

[21] Su D.-L., Li P. J., Quek S. Y. (2019). Efficient extraction and characterization of pectin from orange peel by a combined surfactant and microwave assisted process. *Food Chemistry*.

[22] Priyangini F., Walde S. G., Chidambaram R. (2018). Extraction optimization of pectin from cocoa pod husks (*Theobroma cacao L*.) with ascorbic acid using response surface methodology. *Carbohydrate Polymers*.

[23] Vriesmann L. C., Teófilo R. F., Lúcia de Oliveira Petkowicz C. (2012). Extraction and characterization of pectin from cacao pod husks (*Theobroma cacao L*.) with citric acid. *LWT*.

[24] Hosseini S. S., Khodaiyan F., Saeid Yarmand M. (2016). Optimization of microwave assisted extraction of pectin from sour orange peel and its physicochemical properties. *Carbohydrate Polymers*.

[25] Rahmati S., Abdullah A., Kang O. L. (2019). Effects of different microwave intensity on the extraction yield and physicochemical properties of pectin from dragon fruit (Hylocereus polyrhizus) peels. *Bioactive Carbohydrates and Dietary Fibre*.

[26] Gerhardt A. H. (2009). Moisture effects on solid dosage forms--formulation, processing, and stability. *Journal of GXP Compliance*.

[27] Emery E., Oliver J., Pugsley T., Sharma J., Zhou J. (2009). Flowability of moist pharmaceutical powders. *Powder Technology*.

[28] Akpabio E., Jackson C., Ubulom P. (2011). Formulation and in vitro release properties of va plant gum obtained from Sesamum indicum (fam. Pedaliaceae). *International Journal of Pharmaceutical and Biomedical Research*.

[29] USP (2007). *The United States Pharmacopeia, the National Formulary*.

[30] Matsui K., Tsume Y., Amidon G. E., Amidon G. L. (2015). In vitro dissolution of fluconazole and dipyridamole in gastrointestinal simulator (GIS), predicting in vivo dissolution and drug–drug interaction caused by acid-reducing agents. *Molecular Pharmaceutics*.

[31] Hofmann M., García M. A., Al-Gousous J. (2020). In vitro prediction of in vivo absorption of ibuprofen from suspensions through rational choice of dissolution conditions. *European Journal of Pharmaceutics and Biopharmaceutics*.

[32] Dong W. Y., Maincent P., Bodmeier R. (2007). In vitro and in vivo evaluation of carbamazepine-loaded enteric microparticles. *International Journal of Pharmaceutics*.

[33] Schmidt C., Bodmeier R. (2001). A multiparticulate drug-delivery system based on pellets incorporated into congealable polyethylene glycol carrier materials. *International Journal of Pharmaceutics*.

[34] Dey N. S., Majumdar S., Rao M. E. B. (2008). Multiparticulate drug delivery systems for controlled release. *Tropical Journal of Pharmaceutical Research*.

[35] Carbinatto F. M., Dóris de Castro A., Cesar Evangelista R., Ferreira Cury B. S. (2014). Insights into the swelling process and drug release mechanisms from cross-linked pectin/high amylose starch matrices. *Asian Journal of Pharmaceutical Sciences*.

[36] Lee H., Xu G., Kharaghani D. (2017). Electrospun tri-layered zein/PVP-GO/zein nanofiber mats for providing biphasic drug release profiles. *International Journal of Pharmaceutics*.

[37] Jha M. K., Rahman H., Rahman M. (2011). Biphasic oral solid drug delivery system: a review. *International Journal of Pharmaceutical Sciences and Research*.

[38] Rao N. R., Hadi M. A., Panchal H. (2012). Formulation and evaluation of biphasic drug delivery system of Montelukast sodium for chronotherapy. *World Journal of Pharmaceutical Research*.

[39] Yu D. G., Wang X., Li X. Y., Chian W., Li Y., Liao Y. Z. (2013). Electrospun biphasic drug release polyvinylpyrrolidone/ethyl cellulose core/sheath nanofibers. *Acta Biomaterialia*.

[40] Liu L., Fishman M. L., Kost J., Hicks K. B. (2003). Pectin-based systems for colon-specific drug delivery via oral route. *Biomaterials*.

[41] Liu L., Fishman M. L., Hicks K. B. (2007). Pectin in controlled drug delivery–a review. *Cellulose*.

[42] Sriamornsak P. (2011). Application of pectin in oral drug delivery. *Expert Opinion on Drug Delivery*.

[43] Wu B., Chen Z., Wei X., Sun N., Lu Y., Wu W. (2007). Biphasic release of indomethacin from HPMC/pectin/calcium matrix tablet: I. Characterization and mechanistic study. *European Journal of Pharmaceutics and Biopharmaceutics*.

[44] Wu B., Deng D., Lu Y., Wu W. (2008). Biphasic release of indomethacin from HPMC/pectin/calcium matrix tablet: II. Influencing variables, stability and pharmacokinetics in dogs. *European Journal of Pharmaceutics and Biopharmaceutics*.

[45] Nykänen P., Lempää S., Aaltonen M. L., Jürjenson H., Veski P., Marvola M. (2001). Citric acid as excipient in multiple-unit enteric-coated tablets for targeting drugs on the colon. *International Journal of Pharmaceutics*.

[46] Ullah M., Ullah H., Murtaza G., Mahmood Q., Hussain I. (2015). Evaluation of influence of various polymers on dissolution and phase behavior of carbamazepine-succinic acid cocrystal in matrix tablets. *BioMed Research International*.

[47] Mohammed F. A. (2012). Formulation and evaluation of carbamazepine extended release tablets USP 200 mg. *International Journal of Biological & Pharmaceutical Research*.

[48] Dzajkowska M., Hanna K., Anna M. (2017). Prolonged‐release minitablets with carbamazepine–preliminary observations in vitro. *Journal of Pharmacy and Pharmacology*.

[49] Imam S. S., Aqil M., Akhtar M., Sultana Y., Ali A. (2015). Formulation by design-based proniosome for accentuated transdermal delivery of risperidone: in vitro characterization and in vivo pharmacokinetic study. *Drug Delivery*.

[50] Imam S. S., Ahad A., Aqil M., Akhtar M., Sultana Y., Ali A. (2017). Formulation by design based risperidone nano soft lipid vesicle as a new strategy for enhanced transdermal drug delivery: in-vitro characterization, and in-vivo appraisal. *Materail Science and Engineering*.

[51] Nettey H., Allotey-Babington G. L., Somuah I. (2017). Assessment of formulated amodiaquine microparticles in Leishmania Donovani infected rats. *Journal of Microencapsulation*.

[52] Khan N., Shah F. A., Rana I. (2020). Nanostructured lipid carriers-mediated brain delivery of carbamazepine for improved in vivo anticonvulsant and anxiolytic activity. *International Journal of Pharmaceutics*.

